# 2-Benzamido-*N*-(1*H*-benzo[*d*]imidazol-2-yl)thiazole-4-carboxamide derivatives as potent inhibitors of CK1δ/ε

**DOI:** 10.1007/s00726-012-1234-x

**Published:** 2012-02-14

**Authors:** Joachim Bischof, Johann Leban, Mirko Zaja, Arnhild Grothey, Barbara Radunsky, Olaf Othersen, Stefan Strobl, Daniel Vitt, Uwe Knippschild

**Affiliations:** 1 Department of General, Visceral and Transplantation Surgery, Surgery Centre, University of Ulm, Steinhövel Str. 9, 89075 Ulm, Germany; 2 4SC AG, Am Klopferspitz 19a, 82152 Planegg-Martinsried, Germany; 3 Present Address: Division of Surgery, Oncology, Reproductive Biology and Anaesthetics Hammersmith Campus, Imperial College, Cyclotron Building, 5th Floor, London, W12 0NN UK

**Keywords:** CK1δ, CK1ε, Phosphorylation, Small molecule inhibitor, Crystal structure, MTT, FACS

## Abstract

In this study we identified two heterocyclic compounds (**5** and **6**) as potent and specific inhibitors of CK1δ (IC_50_ = 0.040 and 0.042 μM, respectively). Whereas compound **5** exhibited fivefold higher affinity towards CK1δ than to CK1ε (IC_50_ CK1ε = 0.199 μM), compound **6** also inhibited CK1ε (IC_50_ = 0.0326 μM) in the same range as CK1δ. Selected compound **5** was screened over 442 kinases identifying **5** as a highly potent and selective inhibitor of CK1δ. X-ray analysis of **5** bound to CK1δ demonstrated its binding mode. In addition, characterization of **5** and **6** in a cell biological approach revealed the ability of both compounds to inhibit proliferation of tumor cell lines in a dose and cell line specific manner. In summary, our optimizations lead to the development of new highly selective CK1δ and ε specific inhibitors with biological activity.

## Introduction

Protein kinases in general represent attractive targets for drug development. Recently, interest in specifically targeting members of the casein kinase 1 (CK1) family, a highly conserved ubiquitously expressed serine/threonine protein kinase family, has increased enormously (Knippschild et al. 2005a). The seven mammalian isoforms CK1α, β, γ_1_, γ_2_, γ_3_, δ and ε and their various splice variants are all highly conserved within their kinase domains (~290 residues), but differ significantly within their regulatory N-terminal and C-terminal domains. CK1 isoforms can be regulated by inhibitory autophosphorylation mainly occurring within their C-terminal domains, site-specific phosphorylation mediated by cellular kinases, dephosphorylation of autophosphorylation sites, cleavage of the C-terminal domain, and subcellular compartmentalization (Giamas et al. 2007; Knippschild et al. 2005a). CK1 isoforms phosphorylate many different substrates bearing either a canonical or a non-canonical consensus sequence. They are involved in the regulation of many different cellular processes such as canonical Wnt signaling, DNA damage response, cell cycle progression, apoptosis and chromosome segregation (Cheong and Virshup 2010; Price 2006; Knippschild et al. 2005a, b). Since deregulation of CK1 isoforms have been linked to the development of various types of disorders such as cancer (CK1α/γ/δ/ε), neurodegenerative diseases (CK1δ), and inflammatory disorders (CK1α/δ/ε), the use of CK1 (isoform)-specific inhibitors may have therapeutic potential in the cure of these diseases (Gill et al. 2007; Knippschild et al. 2005a; Lin and Peng 2006; Perez et al. 2010).

So far, several CK1-specific inhibitors have been identified, among them IC261 and D4476 (Mashhoon et al. 2000; Rena et al. 2004). However, permeability to the cell membrane for most available CK1 inhibitors is weak, and their use in vivo restricted. Therefore, efforts are ongoing to identify new potential CK1-specific inhibitors with IC_50_ values in the low nanomolar range which can be used in pharmacological studies and might be effective as therapeutic drugs.

Previously we identified piperidinyl-thiazoles as inhibitors of nuclear factor kappa B (NFκB) (Leban et al. 2007). The well-characterized NFκB is a key player in the signal transduction of severe diseases such as muscular dystrophy, obesity, atherosclerosis, cystic fibrosis, arthritis, Crohn’s disease, sepsis, rheumatic disease and cancer (Baghdiguian et al. 1999; Li et al. 2008; Peterson and Guttridge 2008; Bamborough et al. 2010; Demer and Tintut 2011; Nichols et al. 2008; Criswell 2010; Li et al. 2009; Wei and Feng 2010; Gil et al. 2007).When we deleted the piperidinyl residue the compound series described here was obtained. This series exhibited only modest NFκB inhibitory activity (data not shown) but showed significant inhibition of CK1 family members in a selectivity screen comprising 442 eukaryotic protein kinases.

## Materials and methods

### Intermediate synthesis

#### Ethyl 2-(2-(trifluoromethoxy)benzamido)thiazole-4-carboxylate

To a solution of ethyl 2-aminothiazole-4-carboxylate (12 g, 70 mmol, 1 eq.) in dry THF (300 ml) *N*,*N*-diisopropylethylamine (250 ml, 209 mmol, 3 eq.) was added. A solution of 2-(trifluoromethoxy)-benzoyl chloride (19 g, 84 mmol, 1.2 eq.) in THF (50 ml) was added dropwise at 0°C. The reaction mixture was stirred for 24 h at room temperature. Water (50 ml) was added and THF was removed under reduced pressure. The residue was extracted with DCM. The organic layer was dried over MgSO_4_, filtered and concentrated under reduced pressure. The residue was purified by flash column chromatography on silica gel (PE/EtOAc 80:20). The product was obtained as a white solid (12 g, 33 mmol, 48% yield).

^1^H NMR (400 MHz, DMSO-d_6_): δ (ppm) = 1.30 (t, *J* = 7.1 Hz, 3H), 4.29 (*q*, *J* = 7.1 Hz, 2H), 7.49–7.59 (m, 2H), 7.71 (dt, *J* = 7.8, 1.7 Hz, 1H), 7.79 (dd, *J* = 7.79, 1.7 Hz, 1H), 8.14 (s, 1H), 13.11 (bs, 1H).

#### 2-(2-(Trifluoromethoxy)benzamido)thiazole-4-carboxylic acid

Ethyl 2-(2-(trifluoromethoxy)benzamido)thiazole-4-carboxylate (10 g, 28 mmol, 1 eq.) was dissolved in THF (20 ml) and an aqueous 2 M NaOH solution (110 ml) was added at room temperature. The reaction mixture was stirred for 24 h at room temperature. THF was removed under reduced pressure. The residual aqueous phase was acidified to pH 1–2 using a 15% aqueous HCl solution. The precipitate was collected by filtration, washed with water and dried. The product was obtained as a white solid (9.3 g, 28 mmol, quant.).

^1^H NMR (400 MHz, DMSO-d_6_): δ (ppm) = 7.49–7.59 (m, 2H), 7.70 (dt, *J* = 7.8, 1.7 Hz, 1H), 7.79 (dd, *J* = 7.79, 1.7 Hz, 1H), 8.06 (s, 1H), 13.01 (bs, 2H).

### Final compounds

#### Methyl 2-(2-(2-(trifluoromethoxy)benzamido)-thiazole-4-carboxamido)-1*H*-benzo[*d*]imidazole-5-carboxylate (**1**)

*N*,*N*-diisopropylethylamine (6.1 g, 47.1 mmol) was added dropwise to a solution of 2-(2-(trifluoromethoxy)benzamido)thiazole-4-carboxylic acid (5.2 g, 15.7 mmol) in 50 ml DMF. Then HBTU (6.2 g, 16.5 mmol) was added at 0°C and the reaction mixture was stirred for 25 min. Methyl 2-amino-1*H*-benzo[*d*]imidazole-5-carboxylate (3.0 g, 15.7 mmol) was added portionwise and the reaction mixture was stirred for 16 h. Water (250 ml) was added and the reaction mixture was stirred for 20 min. Then a saturated aqueous NaHCO_3_ solution (20 ml) was added and the mixture was stirred for further 20 min. The suspension was filtered and the solid was washed with water and dried. The residue was purified by flash column chromatography on silica (DCM/MeOH 100:0–100:5). All product fractions were collected and concentrated under reduced pressure. The residue was washed with cold MeOH and dried. The product was obtained as a white solid (2.4 g, 4.7 mmol, 30% yield).

^1^H NMR (400 MHz, DMSO-d_6_): δ (ppm) = 3.86 (s, 3H), 7.48–7.64 (m, 3H), 7.73 (dt, *J* = 7.9, 1.5 Hz, 1H), 7.76–7.88 (m, 2H), 8.12 (s, 1H), 8.36 (s, 1H), 11.42 (bs, 1H), 12.54 (bs, 1H), 13.10 (bs, 1H).

#### *N*-(5-(Methylsulfonyl)-1*H*-benzo[*d*]imidazol-2-yl)-2-(2-(trifluoromethoxy)benzamido)thiazole-4-carboxamide (**2**)

2-(2-(Trifluoromethoxy)benzamido)thiazole-4-carboxylic acid (0.30 g, 0,9 mmol), 5-(methylsulfonyl)-1*H*-benzo[*d*]imidazol-2-amine (0.19 g, 0.9 mmol), HBTU (0.34 g, 0.9 mmol) and DMAP (11 mg, 0.09 mmol) were dissolved in DMF (20 ml). The reaction mixture was stirred for 16 h at 60°C. The reaction mixture was diluted with EtOAc and washed with a saturated aqueous NaHCO_3_ solution and a 5% aqueous citric acid solution. The organic phase was dried over MgSO_4_ filtered and concentrated under reduced pressure. The residue was purified by preparative HPLC/MS. Only the pure fractions were collected and concentrated under reduced pressure. The product was obtained as a white solid (16 mg, 0.03 mmol, 3% yield).

#### *N*-(1-Methyl-1*H*-benzo[*d*]imidazol-2-yl)-2-(2-(trifluoromethoxy)benz-amido)thiazole-4-carboxamide (**3**)

To a solution of 2-(2-(trifluoromethoxy)benzamido)thiazole-4-carboxylic acid (3.2 g, 9.7 mmol) in DMF (45 ml) HBTU (3.9 g, 10.2 mmol) was added portionwise at 0°C, followed by dropwise addition of *N*,*N*-diisopropylethylamine (3.8 g, 29.1 mmol). The cooling bath was removed and the reaction mixture was stirred for 10 min. 1-methyl-1*H*-benzo[*d*]imidazol-2-amine (1.5 g, 10.2 mmol) was added portionwise and the reaction mixture was stirred at room temperature for 16 h. An aqueous 1 M NaOH solution was added and the reaction mixture was stirred for 15 min. The mixture was diluted with water (10 ml) and extracted with MTBE. The organic extracts were extracted with an aqueous 1 M NaOH solution. The combined aqueous phases were acidified to pH 1–2 using conc. HCl. The formed precipitate was filtered and washed with PE and dried. The product was obtained as a white solid (3.0 g, 6.5 mmol, 64% yield).

^1^H NMR (400 MHz, DMSO-d_6_): δ (ppm) = 3.69 (s, 3H), 7.16-7.31 (m, 2H), 7.41–7.60 (m, 4H), 7.64–7.75 (m, 1H), 7.76–7.85 (m, 1H), 8.11 (s, 1H), 12.56 (bs, 1H), 13.00 (bs, 1H).

#### 2-(Isonicotinamido)-*N*-(6-(trifluoromethyl)-1*H*-benzo[*d*]imidazol-2-yl)thiazole-4-carboxamide (**4**)

To a solution of 2-(isonicotinamido)thiazole-4-carboxylic acid (0.4 g, 1.6 mmol) and 6-(trifluoromethyl)-1*H*-benzo[*d*]imidazol-2-amine (0.3 g, 1.6 mmol) in DMF (5 ml) HBTU (0.6 g, 1.6 mmol) and *N*,*N*-diisopropylethylamine (0.3 ml, 0.2 g, 1.6 mmol) were added. The reaction mixture was stirred at room temperature for 16 h. All volatiles were removed under reduced pressure. The residue was suspended in water and the solid filtered off. The crude product was purified by preparative HPLC/MS. Only the pure fractions were collected and concentrated under reduced pressure. The product was obtained as a white solid (12 mg, 0.03 mmol, 2% yield).

#### 2-(2-(Trifluoromethoxy)benzamido)-*N*-(6-(trifluoromethyl)-1*H*-benzo[*d*]imidazol-2-yl)thiazole-4-carboxamide (**5**)

To a solution of 2-(2-(trifluoromethoxy)benzamido)thiazole-4-carboxylic acid (3.4 g, 10.4 mmol) in DMF (50 ml) *N*,*N*-diisopropylethylamine (4.0 g, 31.1 mmol) was added dropwise at 0°C followed by the addition of HBTU (4.1 g, 10.9 mmol). After stirring for 15 min at 0°C 6-(trifluoromethyl)-1*H*-benzo[*d*]imidazol-2-amine (2.1 g, 10.4 mmol) was added portionwise. The reaction mixture was stirred at room temperature for 16 h. All volatiles were removed under reduced pressure. The residue was dissolved in MTBE and washed with an aqueous 5% citric acid solution. The organic phase was extracted with an aqueous 1 M NaOH solution. The combined aqueous phases were acidified to pH 1–2 using conc. HCl. The formed precipitate was filtered and washed with PE and dried. The product was obtained as a white solid (1.5 g, 2.9 mmol, 28% yield).

^1^H NMR (400 MHz, DMSO-d_6_): δ (ppm) = 7.42–7.49 (m, 1H), 7.51–7.61 (m, 2H), 7.63–7.76 (m, 2H), 7.79–7.86 (m, 1H), 8.38 (s, 1H), 11.38 (bs, 1H), 12.60 (bs, 1H), 13.11 (bs, 1H).

#### *N*-(5-Chloro-6-fluoro-1*H*-benzo[*d*]imidazol-2-yl)-2-(2-(trifluoromethoxy) benzamido)thiazole-4-carboxamide (**6**)

To a solution of 2-(2-trifluoromethoxy-benzoylamino)-thiazole-4-carboxylic acid (3.0 g, 9.0 mmol) in DMF 5-chloro-6-fluoro-1*H*-benzo[*d*]imidazol-2-amine (1.8 g, 9.5 mmol) and *N*,*N*-diisopropylethylamine (4.5 ml, 3.5 g, 27.1 mmol) were added. The reaction mixture was cooled to 0°C and HBTU (3.6 g, 9.5 mmol) was added portionwise. The reaction mixture was stirred at room temperature for 16 h. All volatiles were removed under reduced pressure. The residue was washed with a saturated aqueous NaHCO_3_ solution, a 5% aqueous citric acid solution and MeOH. The residue was purified by flash column chromatography on silica (DCM/MeOH 95:5). The product was obtained as a white solid (1.1 g, 2.2 mmol, 24% yield).

^1^H NMR (400 MHz, DMSO-d_6_): δ (ppm) = 7.47 (d, J = 9.75 Hz, 1H), 7.51–7.60 (m, 2H), 7.63 (d, *J* = 6.87 Hz, 1H), 7.73 (dt, *J* = 7.86, 1.68 Hz, 1H), 7.82 (dd, *J* = 7.56, 1.59 Hz, 1H), 8.37 (s, 1H), 11.24 (bs, 1H), 12.46 (bs, 1H), 13.10 (bs, 1H).

#### 2-(Furan-2-carboxamido)-*N*-(6-(trifluoromethyl)-1*H*-benzo[*d*]imidazol-2-yl)thiazole-4-carboxamide (**7**)

To a solution of 2-(furan-2-carboxamido)thiazole-4-carboxylic acid (80 mg, 0.3 mmol) and 6-(trifluoromethyl)-1*H*-benzo[*d*]imidazol-2-amine (68 mg, 0.3 mmol) HBTU (127 mg, 0.3 mmol) and *N*,*N*-diisopropylethylamine (117 μl, 0.7 mmol) were added and the reaction mixture was stirred at 85°C for 16 h. The reaction mixture was poured into ice water and the formed precipitate filtered off, washed with water and dried. The product was obtained as a white solid (50 mg, 0.1 mmol, 35% yield).

#### *N*-(1*H*-Benzo[*d*]imidazol-2-yl)-2-(2-(trifluoromethoxy)benzamido)thiazole-4-carboxamide (**8**)

To a solution of 2-(2-(trifluoromethoxy)benzamido)thiazole-4-carboxylic acid (2.0 g, 6.0 mmol) in DMF (15 ml) *N*,*N*-diisopropylethylamine (2.3 g, 18.1 mmol) was added dropwise. The reaction mixture was cooled to 0°C and HBTU (2.4 g, 6.3 mmol) was added portionwise. The reaction mixture was stirred at room temperature for 1 h. Then 1*H*-benzo[*d*]imidazol-2-amine (0.8 g, 6.3 mmol) was added portionwise and the reaction mixture was stirred at room temperature for 48 h. An aqueous 1 M NaOH solution (5 ml) was added and the reaction mixture was stirred for 15 min. The mixture was diluted with water (10 ml) and extracted with MTBE. The organic extracts were extracted with an aqueous 1 M NaOH solution. The combined aqueous phases were acidified to pH 1–2 using conc. HCl. The formed precipitate was filtered and washed with PE and dried. The product was obtained as a white solid (1.1 g, 2.4 mmol, 41% yield).

^1^H NMR (400 MHz, DMSO-d_6_): δ (ppm) = 7.09–7.17 (m, 2H), 7.44–7.51 (m, 2H), 7.52–7.61 (m, 2H), 7.73 (dt, *J* = 7.87, 1.77 Hz, 1H), 7.82 (dd, *J* = 7.58, 1.61 Hz, 1H), 8.27 (s, 1H), 11.91 (bs, 2H), 13.05 (bs, 1H).

#### 2-(2-Trifluoromethoxy-benzoylamino)-thiazole-4-carboxylic acid benzothiazol-2-ylamide (**9**)

2-(2-(Trifluoromethoxy)benzamido)thiazole-4-carboxylic acid (0.1 g, 0.3 mmol) was dissolved in DMF (3 ml) and *N*,*N*-diisopropylethylamine (39 mg, 0.3 mmol) was added. The reaction mixture was stirred for 2 min and HBTU (0.1 g, 0.3 mmol) and benzo[*d*]thiazol-2-amine (45 mg, 0.3 mmol) were added. The reaction mixture was stirred at 70°C for 16 h. All volatiles were removed under reduced pressure. The residue was dissolved in EtOAc and washed with a 5% aqueous citric acid solution, a saturated aqueous NaHCO_3_ solution and water. The organic phase was dried over MgSO_4_, filtered and concentrated under reduced pressure. The residue was purified by preparative TLC (DCM/MeOH 95:5). The product was obtained as a white solid (33 mg, 0.07 mmol, 24% yield).

^1^H NMR (400 MHz, DMSO-d_6_): δ (ppm) = 7.34 (dt, *J* = 8.10, 1.14 Hz), 7.47 (dt, *J* = 8.19, 1.23 Hz, 1H), 7.51–7.61 (m, 2H), 7.73 (dt, *J* = 7.95, 1.77 Hz, 1H), 7.79 (d, *J* = 7.98 Hz, 1H), 7.82 (dd, *J* = 7.59, 1.62 Hz, 1H), 8.02 (d, *J* = 7.32 Hz, 1H), 8.44 (s, 1H), 12.07 (bs, 1H), 13.08 (bs, 1H).

#### *N*-(Benzo[*d*]oxazol-2-yl)-2-(2-(trifluoromethoxy)benzamido)thiazole-4-carboxamide (**10**)

To a solution of 2-(2-(trifluoromethoxy)benzamido)thiazole-4-carboxylic acid (1.4 g, 4.2 mmol) in DMF (50 ml) *N*,*N*-diisopropylethylamine (1.6 g, 12.5 mmol) was added dropwise. The reaction mixture was cooled to 0°C and HBTU (1.7 g, 4.4 mmol) was added portionwise. The reaction mixture was stirred at room temperature for 15 min. Then benzo[*d*]oxazol-2-amine (0.6 g, 4.4 mmol) was added portionwise and the reaction mixture was stirred at room temperature for 16 h.

All volatiles were removed under reduced pressure. The residue was dissolved in MTBE and washed with an aqueous 5% citric acid solution and a saturated aqueous NaHCO_3_ solution. The organic phase was dried over MgSO_4_, filtered and concentrated under reduced pressure. The residue was purified by flash column chromatography on silica (DCM/MeOH 100:0 to 99:1). The crude product was dissolved in a small amount MeOH and water was added. The precipitate was dried to afford the product as a white solid (750 mg, 1.7 mmol, 40% yield).

^1^H NMR (400 MHz, DMSO-d_6_): δ (ppm) = 7.27–7.39 (m, 2H), 7.50–7.76 (m, 5H), 7.81 (d, *J* = 7.59 Hz, 1H), 8.30 (s, 1H), 11.48 (bs, 1H), 13.08 (bs, 1H).

### Plasmids

CK1δTV1 and 2 (transcription variants 1 and 2) were PCR amplified from mouse cDNA (5′-primer TV1 and 2: 5′-GGATCCATGGAGCTGAGGGTCGGGAATAG-3′, 3′-primer TV1: 5′-GGATCCTCATCGGTGCACGACAGACTGA-3′, 3′-primer TV2: 5′-GGATCCCTACTTGCCGTGGTGTTCGAAA-3′) and cloned into pcDNA3.1/V5-His© TOPO^®^ TA cloning vector (Invitrogen, Karlsruhe, Germany) before being subcloned into pGEX-2T expression vector via *Bam*HI to generate plasmids pGEX-2T-mouse CK1δTV1 (FP1170) and pGEX-2T-mouse CK1δTV2 (FP1171).

For the expression of wt rat CK1δ and rat CK1δ^M82F^ as glutathione S-transferase fusion proteins the plasmids pGEX-2T-CK1δ (FP449) (Knippschild et al. 1997) and pGEX-2T-CK1δ^M82F^ (FP1153) (Peifer et al. 2009) were used.

Plasmid pGEX-2T-mouse p53^1–64^ was used to express GST-mouse p53^1–64^ (FP267) which was used as substrate in in vitro kinase reactions (Milne et al. 1992).

### Overexpression and purification of glutathione S-transferase fusion proteins

Expression and purification of the GST-fusion proteins GST-mouse p53^1–64^ (FP267), GST-rat CK1δ (FP449), GST-rat CK1δ^M82F^ (FP1153), GST-mouse CK1δTV1 (FP1170), and GST-mouse CK1δTV2 (FP1171) was carried out as described elsewhere (Wolff et al. 2005). Expression of fusion proteins FP1170 and 1171 was either performed at 37°C for 2 h, or at 15°C for 14 h. FP449 and FP1153 were always expressed at 15°C for 14 h.

### In vitro kinase assays

In vitro kinase assays were carried out in the presence of various potential inhibitors of CK1δ at an ATP concentration of 0.01 mM and dimethyl sulfoxide (DMSO) solvent control as described previously (Knippschild et al. 1996). Where indicated higher ATP concentrations (0.05, 0.1, 0.25 and 0.5 mM) were used. The fusion protein GST-mouse p53^1−64^ (FP267) was used as substrate. Recombinant CK1δ kinase domain (CK1δkd, NEB, Frankfurt am Main, Germany), GST-rat CK1δ (FP449), GST-rat CK1δ^M82F^ (FP1153), GST-mouse CK1δTV1, GST-mouse CK1δTV2 and recombinant human CK1ε (Invitrogen, Karlsruhe, Germany) were used as sources of enzyme. Phosphorylated proteins were separated by SDS-PAGE and the protein bands were visualized on dried gels by autoradiography. The phosphorylated protein bands were excised and quantified by Cherenkov counting.

### Phosphopeptide analysis

Phosphopeptide analysis of in vitro labeled proteins was carried out as described previously (Wolff et al. 2005).

### KINOMEscan: high-throughput kinase selectivity profiling

KINOME*scan*™ was performed to determine binding constants of coumpound **5** to 442 eukaryotic kinases by Ambit Biosciences Cooperation, San Diego, USA.

### X-ray analyses

Crelux (CRELUX GmbH, Martinsried, Germany) produced co-crystals of a human truncated mutant casein kinase 1 δ (CK1δ^1−316, R13N^) with compound **5** that diffract to 1.7 Å resolutions at the ESRF synchrotron radiation source and determined the X-ray structure.

Crystals were obtained using sitting drop vapor diffusion setups. The diluted protein solution (1 mg/ml) was incubated at room temperature for 2 h with 15 mM of compound **5** and then concentrated to 13.5 mg/ml. 0.4 μl of protein solution (13.5 mg/ml in 50 mM HEPES, 200 mM NaCl, 1 mM EDTA, 1 mM DTT, 5 mM β-OG, pH 7.5) was mixed with 0.4 μl of reservoir solution (0.1 M NaCl, 1.4 M (NH_4_)_2_SO_4_, 0.1 M bis–tris, pH 5.8) and equilibrated over 60 μl of reservoir solution. Crystals appeared after 1–3 days.

The structure was determined by molecular replacement using the published CK1δ structure (PDB accession code 1CKI) as search model followed up by refinement of this model with REFMAC5 (Collaborative Computational Project 1994; Murshudov et al. 1996). Several rounds of alternating manual rebuilding and refinement resulted in the final model.

### Modeling M82F mutant and docking

Based on the X-ray structure of human CK1δ^1–316, R13N^ with compound **5** the gatekeeper methionine 82 was mutated to phenylalanine 82 and energetically minimized using MOE (MOE (The Molecular Operating Environment) Version 2010.10, software available from Chemical Computing Group Inc. http://www.chemcomp.com).

Both nitrogen-protonated benzimidazol-tautomers [1H] and [3H] of compounds **4**–**6** were docked without crystal water or co-ligands into the wild type CK1δ and M82F mutant. The best-scored docking solutions and corresponding protein were fully optimized in MOE and rescored. The [3H] tautomers were only used in docking.

### Cell lines

Frwt648 cells [generated by SV40-transformation of F111 cells; (Hinzpeter and Deppert 1987)] and mKSA cells [SV40-transformed Balb/c fibroblasts; (Kit et al. 1969)] were grown in Dulbecco’s modified Eagle’s medium (DMEM) supplemented with 5% fetal calf serum (FCS; Biochrom, Berlin, Germany). The fibrosarcoma cell line HT1080 (Rasheed et al. 1974) was grown in DMEM containing 10% FCS and 2 mM glutamine while both the prostate cancer cell line DU-145 (Stone et al. 1978) and the ovary tissue cancer cell line OVCAR-3 (Hamilton et al. 1983) were maintained in RPMI-1640 supplemented with 10% FCS. The pancreatic cancer cell line Colo357 (Morgan et al. 1980) was grown in DMEM/RPMI (1:1) and the colon adenocarcinoma cell line SW480 (Leibovitz et al. 1976) in Leibovitz L-15 medium, both containing 10% FCS and 2 mM glutamine. All media were supplemented with 100 U/ml penicillin and 100 μg/ml streptomycin (Gibco, Karlsruhe, Germany) and all cell lines were grown at 37°C in a humidified 5% carbon dioxide atmosphere.

### Flow cytometry and cell cycle analysis

Subconfluent Frwt648, mKSA, Colo357, OVCAR-3, HT1080, DU-145 and SW480 cells were treated with 2 or 4 μM of compounds **5** or **6** for 48 h. Untreated and DMSO treated cells served as control. Cells were harvested, washed once with phosphate-buffered saline (PBS), and prepared for cell cycle analysis using the “Cycle Test Plus Kit” (BD, San Jose, USA). Cell cycle profiles were obtained using a FACScan flow cytometer and CellQuest software (BD Biosciences, San Jose, USA).

## Results

### Biological activity of new identified compounds

**Fig. 1:**
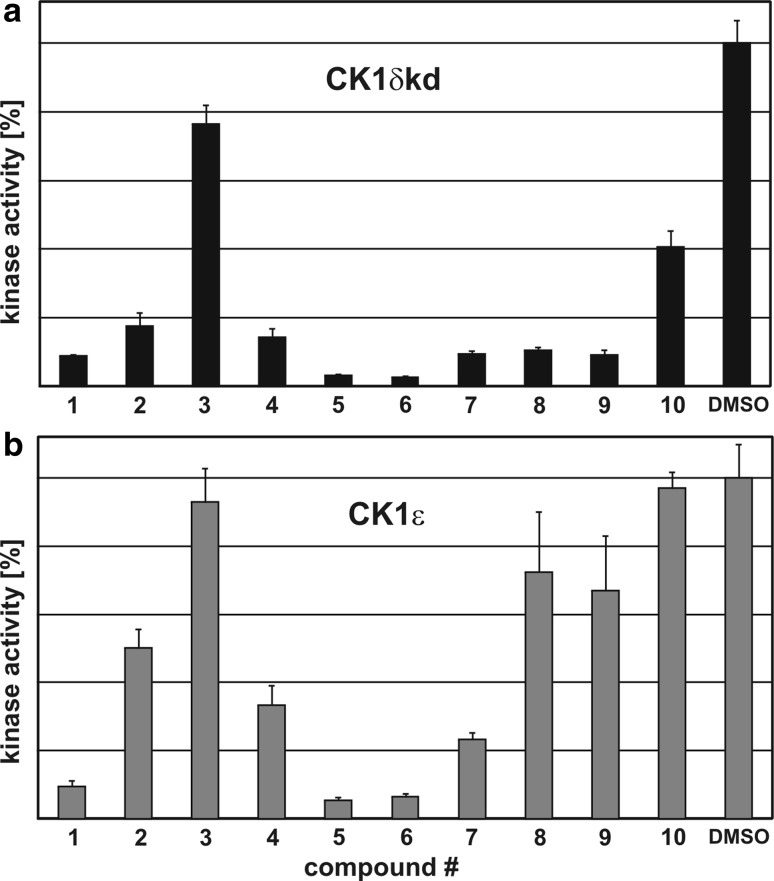
Effect of various inhibitor compounds on CK1δkd and CK1ε kinase activity. Inhibitor compounds **1**–**10** were screened in in vitro kinase assays for biological activity to inhibit CK1δkd **(a)** or CK1ε **(b)**. Each inhibitor was used at a concentration of 10 μM. Results are shown as normalized bar graph

**Fig. 2:**
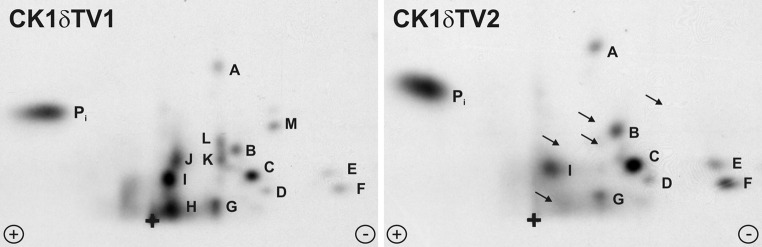
Comparison of the phosphorylation of mouse CK1δTV1 and TV2. Purified GST-mouse CK1δTV1 and GST-mouse CK1δTV2 were autophoshorylated in vitro for 30 min. Phosphorylated proteins were separated by SDS-PAGE, blotted onto PVDF membranes, trypsinized and oxidized. The resulting phosphopeptides were separated in two dimensional analyses by electrophoresis at pH 1.9 and ascending chromatography. Phosphopeptides were labeled with *capital letters*. Phospopeptides *H*, *J*, *K*, *L*, and *M* were only present in CK1δTV1, whereas except *G* and *I* all other phosphopeptides (*A*, *B*, *C*, *D*, *E*, and *F*) of CK1δTV1 were reduced in comparison to those of CK1δTV2

**Fig. 3:**
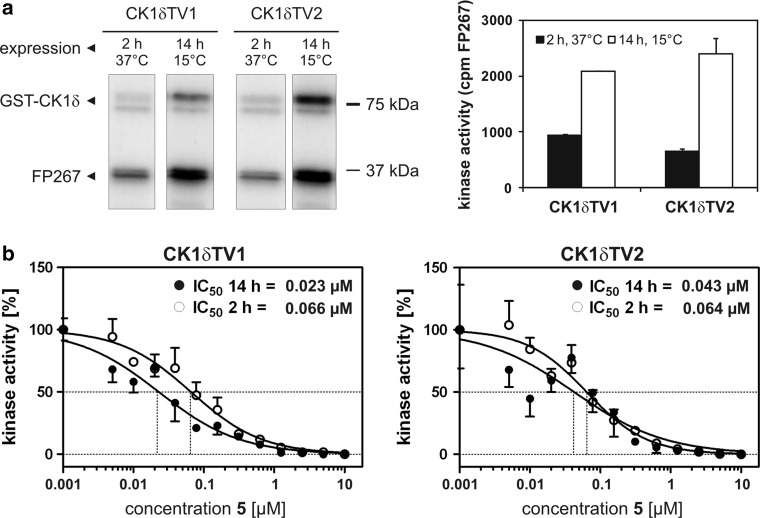
The degree of phosphorylation influences the activity of CK1δ transcription variants and modulates the inhibitory effects of compound** 5**.** a** Comparision of substrate phosphorylation of CK1δ transcription variants induced at 15°C or 37°C. Purified CK1δTV1 and CK1δTV2 either having been induced for 2 h at 37°C or for 14 h at 15°C were used for phosphorylation of GST-mouse p53^1−64^ in vitro. **b** Determination of the inhibitory ability of compound **5** towards CK1δ transcription variants differing in their phosphorylation degree. Compound **5** was tested for its ability to inhibit CK1δTV1 and CK1δTV2 which have either been induced for 2 h at 37°C or for 14 h at 15°C. A high degree of phosphorylation of both CK1δ transcription variants resulted in recuced inhibitory effects of **5** indicated by three- to fourfold higher IC_50_ values

Compounds **1**–**10** (Table 1) were initially assayed for their biological activity against CK1δkd and CK1ε at a concentration of 10 μM ATP (Fig. 1a, b). Compounds **4**, **5** and **6**, which showed significant inhibition of CK1δkd and CK1ε in these assays were further characterized for their IC_50_ values against CK1δkd, GST-rat CK1δ, GST-mouse CK1δTV1, GST-mouse CK1δTV2 and human CK1ε (Table 2). Differences in the IC_50_ values of compounds **4**, **5** and **6** against CK1δ transcription variants could be due to their differences in amino acid composition and the degree of site-specific phosphorylation within their C-terminal regulatory domains. This prediction is underlined by two dimensional phosphopeptide analyses showing quantitative and qualitative differences in the degree of phosphorylation of CK1δTV1 and CK1δTV2 (Fig. 2). Furthermore, induction of recombinant CK1δ transcription variants in bacteria at different temperatures influences its phosphorylation status, activity and sensibility towards small molecule inhibitors (Fig. 3). CK1δ transcription variants induced at 15°C for 14 h are more active and incorporate more radioactive phosphate in the substrate than CK1δ transcription variants induced at 37°C for 2 h (Fig. 3a). Lower degree of phosphorylation of both transcription variants increases the ability of compound **5** to inhibit substrate phosphorylation of both transcription variants indicated by their 1.5- to 2-fold lower IC_50_ values (Fig. 3b).

**Table 1:** CK1 inhibitors

Structure	Compound number
	**1**
	**2**
	**3**
	**4**
	**4b** (only for docking)
	**5**
	**5b** (only for docking)
	**6**
	**6b** (only for docking)
	**7**
	**8**
	**9**
	**10**

**Table 2:** Biological activity of heterocyclic compounds **4**, **5** and **6** against CK1δkd, GST-rat CK1δ, GST-mouse CK1δTV1, GST-mouse CK1δTV2 and CK1ε

No.	IC_50_ (μM) CK1δkd	IC_50_ (μM) GST-CK1δ rat	IC_50_ (μM) GST-CK1δ TV1	IC_50_ (μM) GST-CK1δ TV2	IC_50_ (μM) CK1ε
1	0.865 ± 0.14	1.975 ± 0.85	nd	nd	0.496 ± 0.10
2	1.225 ± 0.41	5.189 ± 1.14	nd	nd	nd
3	>	>	nd	nd	nd
4	0.409 ± 0.12	0.326 ± 0.19	0.180 ± 0.03	0.505 ± 0.04	nd
5	0.029 ± 0.01	0.040 ± 0.01	0.022 ± 0.02	0.042 ± 0.02	0.199 ± 0.15
6	0.085 ± 0.01	0.042 ± 0.05	0.048 ± 0.02	0.055 ± 0.02	0.033 ± 0.01
7	0.542 ± 0.05	1.036 ± 0.31	nd	nd	nd
8	1.116 ± 0.19	0.935 ± 0.55	nd	nd	nd
9	0.730 ± 0.11	1.193 ± 0.39	nd	nd	nd
10	0.647 ± 0.04	0.991 ± 0.21	nd	nd	nd

Results are presented as mean ± SD from experiments performed at least in triplicate

*>*, IC_50_ could not be determined within the range of 10–0.005 μM; *nd*, not determined

### Selectivity profiling of compound **5** in a panel of 442 protein kinases

**Fig. 4:**
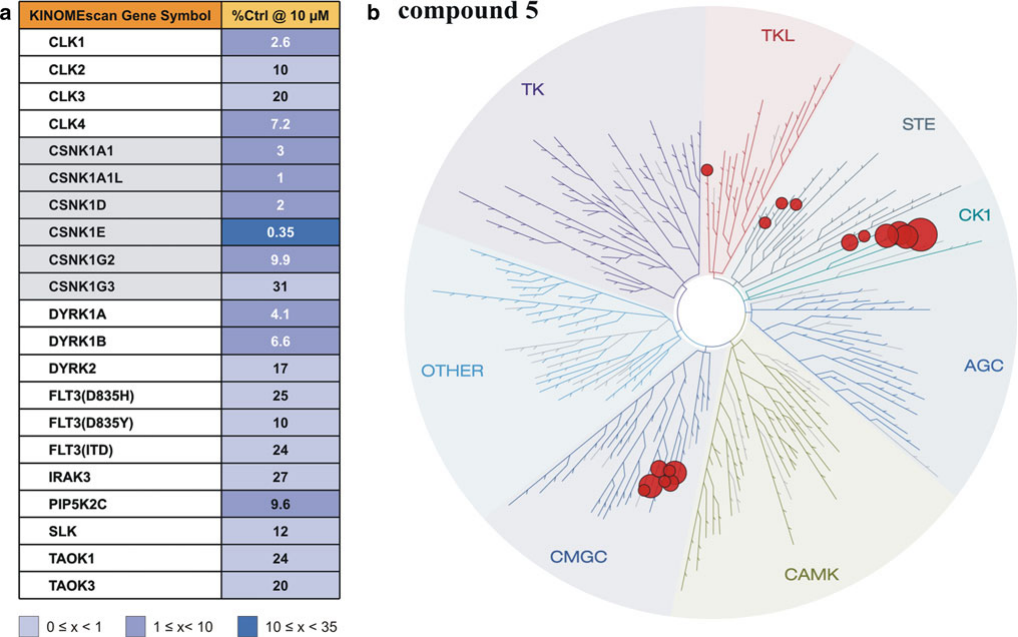
Determination of selectivity of compound **5**. In order to determine target selectivity, a panel of 442 protein kinases was screened for inhibition by compound **5** at a concentration of 10 μM. **a** Targets showing less than 35% of control activity in the presence of **5**. **b** Illustration of targets phylogenetic relations listed in (**a**). Image generated using TREE*spot*™ Software Tool and reprinted with permission from KINOME*scan*™, a division of DiscoveRx Corporation, © DISCOVERX CORPORATION 2010

In order to investigate the specificity of compound **5** its ability to inhibit other kinases at a concentration of 10 μM a KINOME*scan*™ (KINOMEscan, San Diego, USA) with a panel of 442 protein kinases was performed (Fig. 4). In this assay CK1 isoforms were potentially inhibited by compound **5** (CSNK1E 0.35%, CSNK1D 2% and CSNK1A1 3% remaining kinase activity relative to controls), while most kinases were not affected significantly. However, in the presence of compound **5** (10 μM) the kinase activities of few kinases were similarly low, among them CLK1 (2.6%), DYRK1A (4.1%), CLK4 (7.2%), DYRK1B (6.6%), and PIP5K2C (9.6%).

### Binding mode of compound **5** to CK1δ

**Fig. 5:**
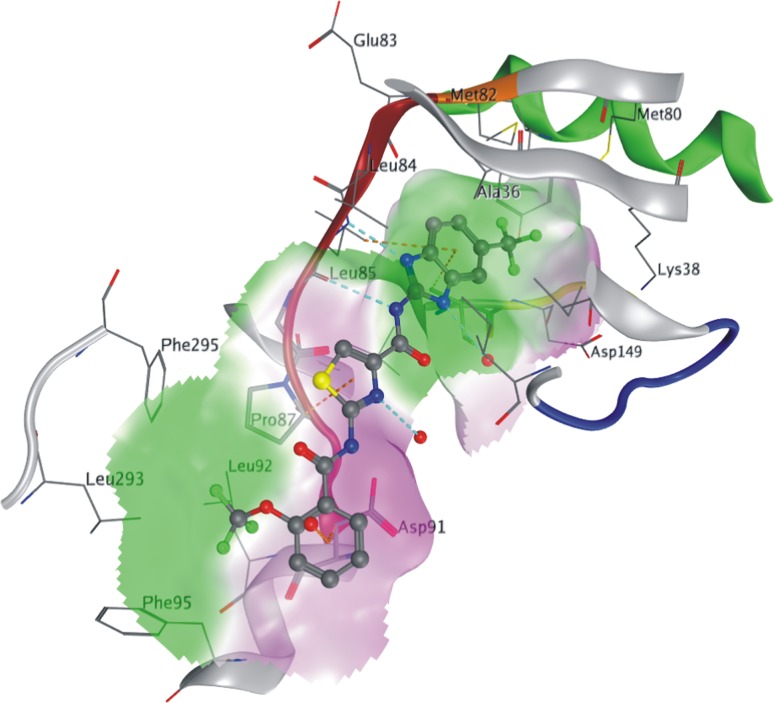
X-ray structure of 5 in the ATP binding pocket of CK1δ. Residues within 4.5 Å of compound **5** are fully shown, whereas the backbone is visualized in parts, color-coded for the kinase-typical structural elements (αC *green*, gatekeeper *orange*, hinge-region *red*, gylcine-rich loop *blue* and DFG-motif *yellow* (*background*)). The *doted lines* depict hydrogen bonds in cyan for standard and orange for π-hydrogen bonds

The tertiary structure of the protein is well conserved in comparison with 1CKI. The DFG-motiv exhibits the ‘in’-conformation (Pargellis et al. 2002). Compound **5** binds to the ATP-site as depicted in Fig. 5. The sidechain conformation of gatekeeper methionine 82 is more compact to accommodate the trifluoromethyl-group but no deeper/selectivity pocket is opened. Hinge residues leucine 84 and leucine 85 are hydrogen bonded via the backbone carbonyl to NH and the backbone nitrogen to the imidazole nitrogen of compound **5**, respectively. CH/π-bonds are formed for all three aromatic regions of compound **5**. Hydrophobic interactions exist between the trifluoromethoxy-group and the sidechains of proline 87, leucine 92, phenylalanine 95, leucine 293 and phenylalanine 295. Furthermore, three crystal water molecules are found near compound **5** saturating hydrogen bond functions of the ligand and the protein surface. These water molecules are all solvent exposed and should therefore have no influence on the binding mode.

### Compounds **4**, **5** and **6** are ATP competitive inhibitors of CK1δ

**Fig. 6:**
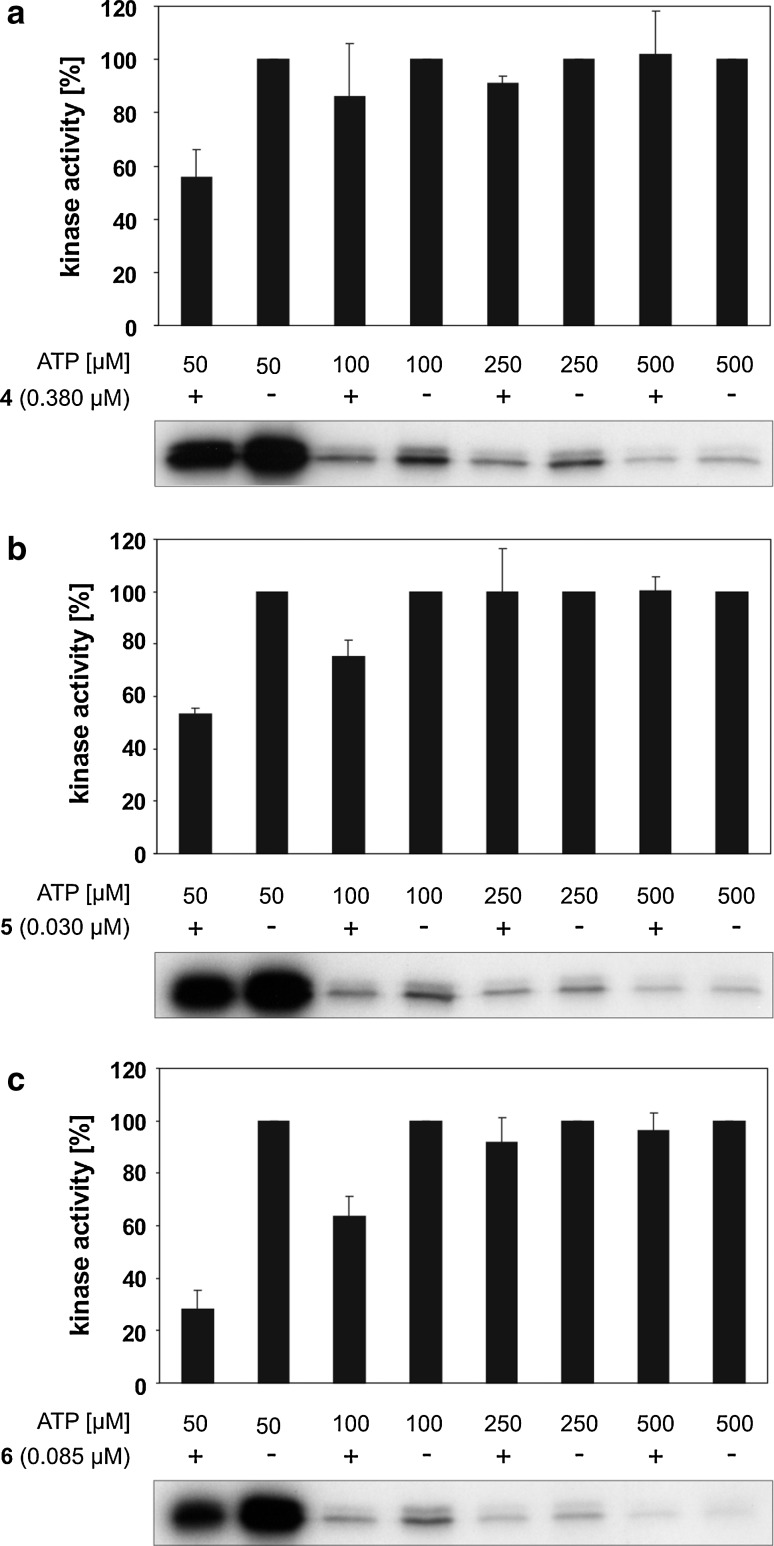
Compounds **4, 5** and **6** inhibit CK1δ in an ATP competitive manner. Inhibitors **4** (**a**; 380 nM), **5** (**b**; 30 nM) and **6** (**c**; 85 nM) were assayed in the presence of the indicated ATP concentrations. Kinase assays were perfomed using CK1δkd as enzyme and GST-p53^1−64^ fusion protein (FP267) as substrate. Kinase activity in reactions containing inhibitor was calculated relative to the control reaction for each ATP concentration. While ATP concentrations increase, incorporation of radioactive labeled phosphate into substrate FP267 decreases, leading to weakened signals in the autoradiographs

In order to prove these compounds as ATP competitive inhibitors, **4**, **5** and **6** were tested at their IC_50_ concentrations for the potency to inhibit CK1δkd in the presence of different amounts of ATP (Fig. 6a–c). Since the IC_50_ values increased progressively upon raising the concentration of ATP the ATP competitive properties of all tested compounds were confirmed. This finding underlines and clearly shows that **4**, **5** and **6** are highly potent inhibitors of CK1δ which are able to bind and block kinase activity even in the presence of increased ATP concentrations.

### Inhibitory effects of compounds **4**, **5** and **6** on GST-wt CK1δ and GST-CK1δ^M82F^

**Fig. 7:**
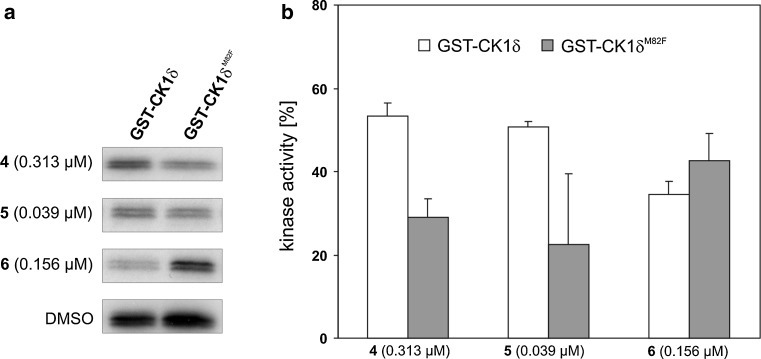
Inhibition of GST-wt CK1δ and a GST-CK1δ^M82F^ gatekeeper mutant**. a** Compounds **4** (0.313 μM), **5** (0.039 μM) and **6** (0.156 μM) were assayed for their ability to inhibit GST-wt CK1δ in comparison to a GST-CK1δ^M82F^ gatekeeper mutant using GST-p53^1−64^ fusion protein (FP267) as substrate. GST-CK1δ^M82F^ shows stronger inhibition of kinase activity in the presence of compounds **4** and **5** and a lower inhibition in the presence of compound **6** than GST-wt CK1δ. **b** Kinase activity is presented as bar graph normalized towards solvent controls

Previously it has been shown that methionine 82 plays an important role as gatekeeper residue in the docking mode of isoxazoles to the ATP binding pocket since mutation of methionine 82 to phenylalanine blocks binding of this class of CK1δ specific inhibitors (Peifer et al. 2009) while still binding ATP. Therefore, we now analyzed the effects of exchanging methionine 82 to phenylalanine on the ability of compounds **4**, **5** and **6** to inhibit CK1δ activity. In vitro kinase assays were performed in the absence and presence of **4, 5** and **6** at their determined IC_50_ concentrations using GST-wt CK1δ or GST-CK1δ^M82F^ as the source of enzyme. GST-wt CK1δ activity was clearly decreased in the presence of **4**, **5** and **6**. Interestingly, in comparison with inhibition of GST-wt CK1δ the kinase activity of GST-CK1δ^M82F^ was much more affected in reactions containing compounds **4** or **5**, but similarly or even less affected by compound **6** (Fig. 7). These observations underline the different binding mode of these compounds than that of isoxazoles, which address the selectivity pocket, while compounds **4**–**6** do not bind to this region in the active site.

### Differences in ligand interaction of compounds **4**, **5** and **6** in wt CK1δ and CK1δ^M82F^

**Fig. 8:**
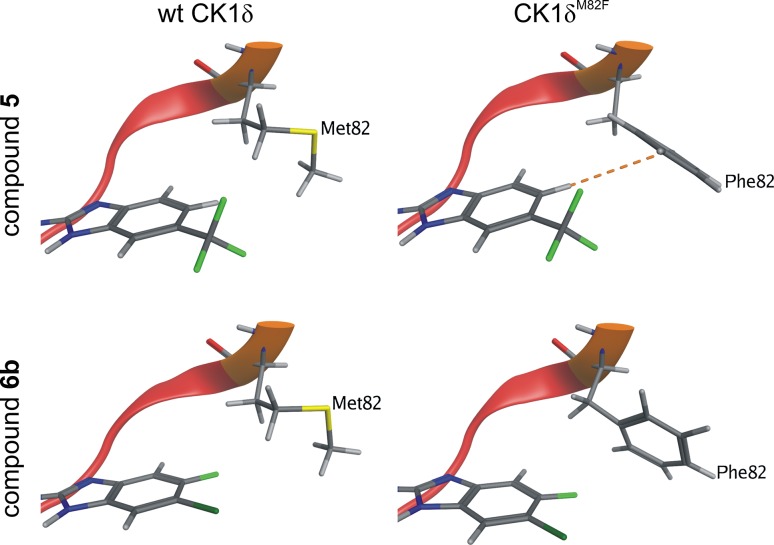
Interaction differences of **5** and **6b** in wt CK1δ and the CK1δ^M82F^ gatekeeper mutant Exemplary docking poses of compounds **5** (*top*) and **6b** (*bottom*) in wt CK1δ (*left*) and CK1δ^M82F^ (*right*). The hinge-region is marked *red* and the gatekeeper *orange*. The π-hydrogen bonds discerning the compounds in inihibition strength are *dotted orange*

Docking poses in wt CK1δ and CK1δ^M82F^ align well with the X-ray determined pose of compound **5** in the wild type. Mutation of methionine 82 has nearly no influence on the docking pose, although the cavity for the ligand is marginally reduced. For compounds **4** and **5** the synthesized [1H] benzimidazole tautomers exhibit a better docking score than the [3H] tautomers **4b** and **5b**, whereas the [3H] tautomer **6b** scores better than the synthesized compound **6**. As tautomerism within the assay cannot be excluded, both tautomers for compound **6** were optimized and rescored. Regardless of tautomerism, the NH_Leu85_···N_Benzimidazol_ hydrogen bond is always formed (Fig. 5), resulting in a flip of the benzimidazole ring and thus a different orientation of the attached functional groups. However, in all cases the docking scores for compounds **4** and **5** in CK1δ^M82F^ improve compared to wt CK1δ whereas compounds **6** and **6b** fall off and are thus in accordance with the experimental results (Table 3). The differences can be explained by the π-hydrogen bond between benzimidazole and phenylalanine 82, which is not possible for both tautomers of compound **6**. The necessary hydrogen is substituted with fluorine or chlorine, respectively (Fig. 8).

**Table 3:** Docking free energy estimation for optimized and rescored protein-compound complexes in kcal/mol

Compound no.	**4**	**5**	**6**	**6**b
wt CK1δ	−30.2	−32.8	**−32.9**	**−34.1**
CK1δ^M82F^	**−31.2**	**−33.3**	−30.2	−32.8

### Efficacy of **5** and **6** in cell culture

**Fig. 9:**
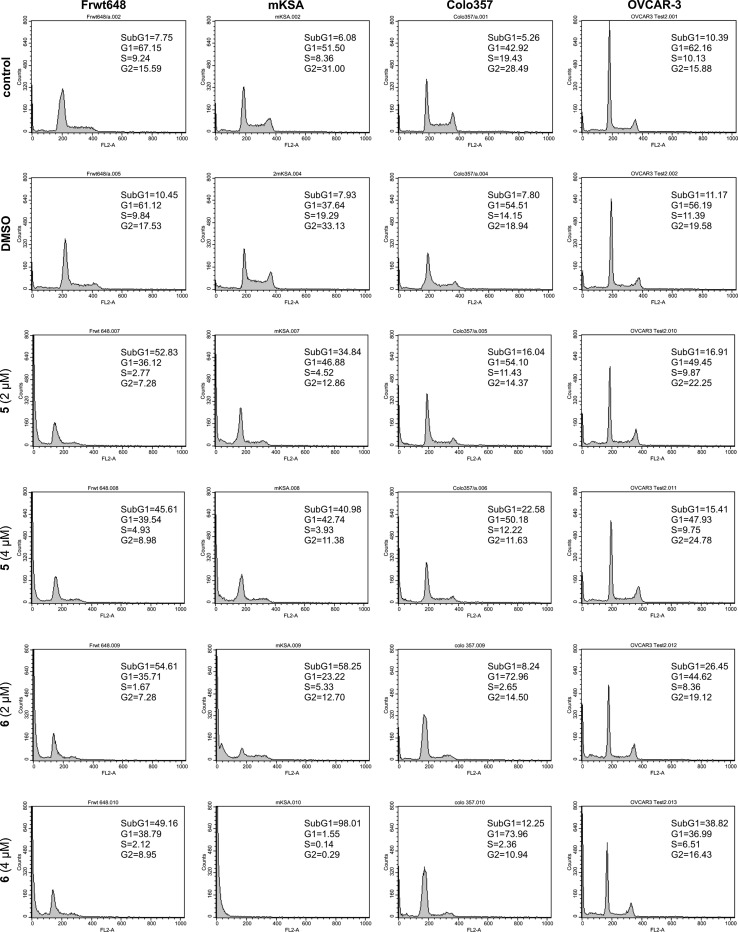
Cell cycle analysis of selected cell lines after treatment with compounds **5** and** 6** Cell cycle analysis of Frwt648, mKSA, Colo357 and OVCAR-3 cells treated with compounds **5** and **6** (2 or 4 μM) for 48 h. Cells were stained with propidium iodide and analyzed using a flow cytometer. Control cells were treated with DMSO. The SV40-transformed Frwt648 and mKSA cells were highly sensitive towards treatment with **5** and **6**. Additionally to the increased amount of dead cells treatment of Colo357 also resulted in more cells in G1 phase of the cell cycle and treatment of OVCAR-3 also slightly increased the amount of cells in G2 phase

Although potent inhibition of CK1δ has been observed for several inhibitor compounds in vitro, these might not necessarily show similar effects in in vivo experiments. In order to identify inhibitors which are able to pass cell membranes and inhibit proliferation of tumor cell lines, a panel of seven cell lines (Frwt648, mKSA, Colo357, OVCAR-3, HT1080, DU-145 and SW480) was either treated with 2 or 4 μM of compounds **5** or **6** (or with DMSO as a negative control) for 48 h. FACS analyses were performed to compare the effects of both compounds with those of vehicle only (DMSO) with respect to cell viability and cell cycle distribution. Our results indicated that the SV40-transformed Frwt648 and mKSA cell lines are highly sensitive towards treatment with 2 and 4 μM of compounds **5** and **6** (35–98% dead cells; Fig. 9). Similar, but significantly weaker effects could be observed for cell lines HT1080, DU-145 and SW480 after 48 h treatment with compounds **5** and **6** (data not shown). However, no measurement using these three cell lines detected more than 20% of dead cells with DU-145 even being unsusceptible to treatment with compound **5** (data not shown). In addition to the increased amount of dead cells after 48 h treatment of Colo357 cells with compounds **5** and **6**, more cells appeared to be in the G1 phase of the cell cycle (Fig. 9). OVCAR-3 cells were more sensitive to compound **6** in the tested concentrations as 22, and 39%, respectively, of the cells died upon treatment. Treatment of OVCAR-3 cells with compound **5** resulted in a slight increase of cells in the G2 phase of the cell cycle (Fig. 9).

In general, results of this screening show cell line specific differences in the potency of the tested inhibitors to induce apoptosis or cell cycle arrest.

## Discussion

Recently, it has become practice to screen cellular pathways in whole cell systems with chemical libraries and then find the cellular target by proteomics or biochemical methods. We have screened for NFκB inhibitors and found hits with nanomolar activity. These compounds were piperidinothiazole carboxylamido-benzimidazoles (Leban et al. 2007). The NFκB pathway is complex and contains many possible targets for inhibition. We therefore further investigated the mechanism of action of these compounds and established that the inhibition of NFκB is derived from the multiple kinase inhibition profile.

To further improve the physicochemical properties of the series we deleted the piperidino part of the molecule and derived at acylaminobenzothiazolocarboxamidobenzothiazoles as exemplified by compounds **1–10** in Table 1.

The compounds were tested in kinase assays using a CK1 specific substrate as described. The parent compound **9** had only moderate but significant effects on CK1δkd (IC_50_ = 1.116 μM). When the benzimidazole NH was replaced by S (as in compound **10)** or O (as in compound **11**), kinase activity was even more decreased. When the NH of the benzimidazole was blocked by methylation in compound **3** activity against CK1δkd was lost. An improvement of activity was obtained when a hydrophobic trifluoromethyl residue was introduced into the benzimidazole in compound **5** (IC_50_ CK1δTV1 = 0.022 μM). Similarily, hydrophobic halogen residues in **6** lead to good activity with an IC_50_ of 0.048 μM for CK1δTV1. If one compares compound **5** with compounds **4** and **7** it is obvious that the trifluoromethoxyphenylacyl on the aminobenzothiazole is optimal. Hydrophylic groups on the benzimidazole as in compound **1** and **2** lead to diminished activity. The SAR presented is in good agreement with the X-ray structure results and fully explains most interactions found.

Although some isoform selective effects of the tested molecules could be observed, especially for compound **5** (up to 7-fold more active on CK1δkd compared to CK1ε), in the concentration range which is commonly used and necessary for cell-based screening and therapeutic application, isoform selectivity will not be observed. Being highly conserved within the kinase domain, the CK1 isoforms significantly differ in their N- and C-terminal domains. According to our results inhibitory (auto-)phosphorylation within the C-terminal regulatory domain not only influences kinase activity but also the effect of inhibitor molecules. When kinase proteins are expressed for 14 h at 15°C C-terminal phosphorylation is reduced leading to increased kinase activity and more potent inhibitor action. Increased C-terminal phosphorylation comes along with less potent action of inhibitor molecules. This observation could be explained by inhibitor compounds competing with the C-terminal domain which can act as pseudo-substrate thereby possibly blocking the catalytic center of the kinase (Gietzen and Virshup 1999; Rivers et al. 1998).

According to the data obtained from the selectivity profiling also CK1 isoforms α and γ turned out to be targets of at least compound **5**. However, effects of the molecules presented in this study have not yet been tested on CK1α and γ.

For the three most effective compounds the postulated binding to the CK1δ protein was validated in vitro. The first approach clearly confirmed the ATP competitive properties of the tested compounds since inhibitory effects are disappearing along with increasing ATP concentration. The second approach, using the CK1δ^M82F^ gatekeeper mutant, is some more sophisticated. The ATP binding site is highly conserved among most protein kinases. However, access of the ligand to the hydrophobic binding pocket can be tightly controlled by various gatekeeping amino acid residues. If these residues get mutated, substrate phosphorylation and also inhibitor action can be affected (Elphick et al. 2007). In the case of rat CK1δ the gatekeeping residue is methionine 82 which was mutated to the more bulky phenylalanine in order to “close the gate” to the kinase’s selectivity pocket (Oumata et al. 2008; Peifer et al. 2009). According to our predictions and X-ray models, the tested compounds are not occupying the selectivity pocket. Therefore, in spite of the “closed gate”, **4** and **5** inhibited the activity of CK1δ^M82F^ better than that of wt CK1δ because of an additional π-hydrogen bond to phenylalanine 82.

In a final biological screen we demonstrated, that compounds **5** and **6** are able to negatively affect the proliferation of several tumor cell lines. Although our cell cycle analysis shows significant effects, we have to take into account that isoforms of the CK1 family of protein kinases are involved in numerous cellular signalling pathways. Thus consideration of the cellular background is crucial for evaluation of any results. In order to get a more detailed insight to affected cellular pathways and functions, more complex approaches for cell culture based profiling are needed.

In conclusion we designed and characterized new inhibitor compounds with remarkable selectivity towards CK1δ and ε.
